# Advances in IL–15–Based Cancer Immunotherapy and Divergent Immunological Effects of IL–2 and IL–15 Signaling via the Shared IL–2Rβγ Receptor

**DOI:** 10.3389/fimmu.2026.1791059

**Published:** 2026-04-10

**Authors:** Huiquan Gao, Tao Ma, Qinqin Jiang, Lanfang Gao, Jinfang Li, Shubo Wang, Ziyong Liu, Zhixin Zhang, Gang Wu, Wenxin He, Fuxin Zhou

**Affiliations:** 1 Department of Radiotherapy, The Affiliated Yantai Yuhuangding Hospital of Qingdao University, Yantai, China; 2 Department of Thoracic Surgery, The 970th Hospital of the People’s Liberation Army, Weihai, Shandong, China; 3 Department of Ultrasound, Weihai Municipal Hospital, Weihai, Shandong, China; 4 Department of Gynecology, The Affiliated Weihai Second Municipal Hospital of Qingdao University, Weihai, Shandong, China; 5 Bioinformatics and Molecular Genetics, Faculty of Biology, University of Freiburg, Freiburg, Germany; 6 Department of Thoracic Surgery, Shanghai Pulmonary Hospital, School of Medicine, Tongji University, Shanghai, China; 7 Department of General and Visceral Surgery, Faculty of Medicine, Medical Center - University of Freiburg, Freiburg, Germany

**Keywords:** cancer immunotherapy, CAR-NK cells, CAR-T cells, cytokine engineering, immune checkpoint inhibitor (ICI), immunocytokines, interleukin-15 (IL-15), interleukin-2 (IL-2)

## Abstract

Interleukin-15 (IL-15) has emerged as a central cytokine for next-generation cancer immunotherapy because of its unique ability to sustain the survival, proliferation, and cytotoxic function of memory CD8^+^ T cells and natural killer (NK) cells without promoting the expansion of regulatory T cells (Treg). These properties make IL-15 particularly attractive for achieving durable antitumor immunity, especially in solid tumors where immune persistence remains a major limitation. Although IL-15 shares the same signal-transducing receptor subunits (IL-2R*β* and the common *γ* chain) with interleukin-2 (IL-2), the two cytokines drive fundamentally different CD8^+^ T-cell fates, a distinction that underlies their markedly divergent therapeutic profiles in cancer immunotherapy. In recent years, multiple IL-15-based therapeutic strategies including recombinant IL-15, and IL-15 immunocytokines have entered clinical evaluation, demonstrating potent immune activation with manageable toxicity profiles. Recent clinical progress includes the FDA approval of Nogapendekin alfa inbakicept (N-803), the first IL-15-based immunotherapy approved for cancer treatment, alongside the advancement of other IL-15 superagonists into Phase II trials and growing evidence that IL-15 can enhance the efficacy of immune checkpoint blockade and engineered adoptive cell therapies such as CAR-T cells, CAR-NK cells, *γδ* T cells, and invariant NKT cells. Despite these advances, important challenges remain, including cytokine-associated toxicities, optimal delivery strategies, and the immunosuppressive tumor microenvironment. This review summarizes recent progress in IL-15-based cancer immunotherapy, integrates emerging insights into IL-2R*βγ*-driven CD8^+^ T-cell fate decisions, and discusses key opportunities and challenges for translating IL-15-mediated immune enhancement into durable clinical benefit.

## 1 Introduction

Immunotherapy for cancer has fundamentally revolutionized modern oncological strategies. Compared to conventional chemotherapy and radiation, immunotherapy harnesses the immune system’s intrinsic capacity to recognize and eliminate malignant cells, thereby offering a distinct and often more durable therapeutic approach (1). This paradigm shift has driven the development of a broad spectrum of cancer immunotherapeutic agents, including recombinant immunostimulatory cytokines (e.g., interleukin-2 (IL-2) and interferon IFN-*α*), immune checkpoint inhibitors targeting PD-1/PD-L1, and adoptive cell therapies such as chimeric antigen receptor (CAR)-T cells (2–4). Despite these remarkable advances, significant challenges remain, such as the identification of optimal immunological targets, mitigation of therapy-associated toxicities, and reliable prediction of patient responsiveness. These challenges have prompted extensive investigation into strategies that enhance efficacy while minimizing toxicity in cancer immunotherapy.

Cytokines represent a critical class of immunomodulatory molecules capable of shaping both innate and adaptive immune responses by acting on effector and cytotoxic immune cells. Extensive preclinical studies across diverse animal tumor models have demonstrated potent antitumor activity for multiple cytokines, including IL-2, granulocyte-macrophage colony-stimulating factor (GM-CSF), IL-12, IL-15, IL-18, IL-7, and IL-21. (5, 6). Consequently, IL-2 was the first immunotherapy approved by the United States Food and Drug Administration (FDA) for cancer treatment in 1990s (7). Encouraged by the efficacy of IL-2, a variety of cytokines including granulocyte-macrophage colony-stimulating factor (GM-CSF) and several interleukins such as IL-15, IL-12, IL-18, IL-7, and IL-21, have been evaluated in clinical trials involving patients with advanced malignancies (8). However, despite its early clinical success, the clinical use of IL-2 has been declined largely due to its severe systemic toxicities, most notably vascular leak syndrome (VLS) (9). IL-2 and IL-15 are closely related members of the common *γ*-chain cytokine family and play central roles in regulating T-cell immunity. Both cytokines signal through a shared receptor complex composed of IL-2R*β* (CD122) and the common *γ* chain (CD132), activating overlapping intracellular pathways that control T-cell proliferation, survival, and differentiation. IL-15, originally identified based on its ability to support T-cell growth, was subsequently recognized as a critical regulator of memory CD8^+^ T cells and NK cells, positioning it as a promising alternative to IL-2 for therapeutic immune activation. Despite their shared receptor usage and signaling machinery, IL-2 and IL-15 exert strikingly different effects on T-cell fate and immune homeostasis, a distinction that has profound implications for cancer immunotherapy (10). Compared to IL-15, IL-2 is able to maintain regulatory T cells (Tregs), which can suppress antitumor immune responses (11). Moreover, IL-2 promotes activation-induced cell death (AICD) of CD8^+^ effector T cells, thereby limiting sustained antitumor immunity (8). Consistent with these functional differences, genetic deficiency of IL-2 leads to immune dysregulation and severe autoimmunity (12–15), whereas IL-15 deficiency results in an almost complete absence of NK cells and a profound reduction in effector and memory CD8^+^ T-cell populations (16).

Importantly, preclinical studies have further revealed distinct toxicity profiles between the two cytokines. IL-15 administration is associated with minimal vascular capillary leak syndrome compared to IL-2, suggesting a more favorable therapeutic window (17). Collectively, these biological and functional distinctions support the premise that IL-15 may represent a more efficacious and better-tolerated cytokine for cancer immunotherapy. In this review, we summarize the biology and immunological functions of IL-15, highlight the mechanistic divergence between IL-2- and IL-15-mediated signaling, examine the current landscape of IL-15-based clinical strategies, and discuss future directions for optimizing cytokine-supported immunotherapy in solid malignancies.

## 2 The biology of IL-15 and IL-15 receptors

**Figure 1:**
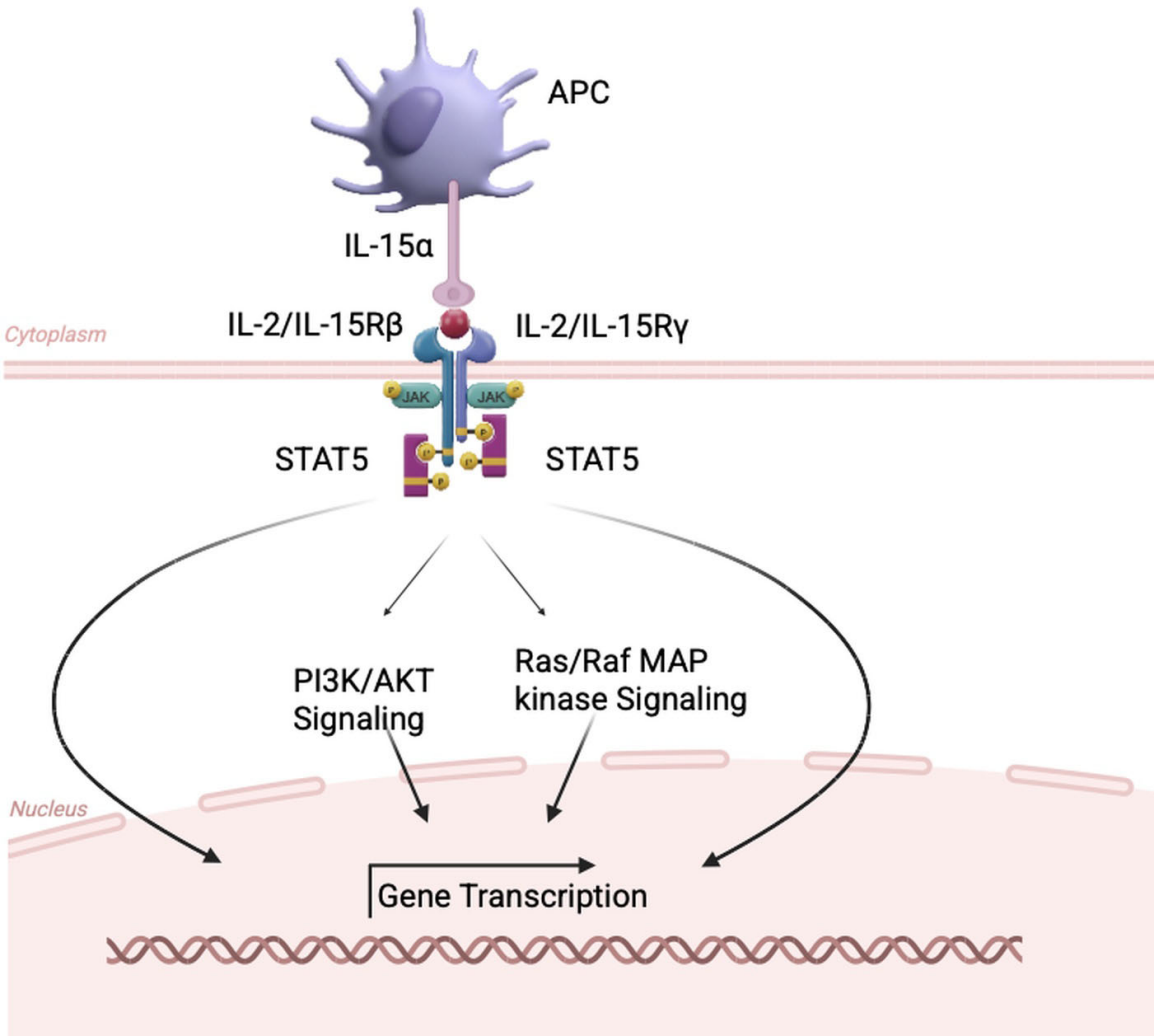
Distinct modes of IL-2 and IL-15 signaling through shared IL-2R*βγ* receptors. IL-2 is secreted as a soluble cytokine and signals through the high-affinity IL-2 receptor complex (IL-2R*αβγ*) expressed on activated T cells and NK cells. In contrast, IL-15 is predominantly presented in trans as a preformed IL-15/IL-15R*α* complex on antigen-presenting cells, engaging IL-2R*βγ* on neighboring NK or T cells. Despite utilizing the same signaling receptor subunits, these distinct delivery modes result in different signaling kinetics and immune cell fates.

IL-15 was independently identified in 1994 by two research groups based on the observation that culture supernatants derived from CV-1/EBNA simian kidney epithelial cells or the human T-cell leukemia virus-1 (HTLV-1)-transformed T-cell line HuT-102 supported proliferation of the IL-2-dependent CTLL-2 T-cell line despite neutralization of IL-2, indicating the presence of a distinct T-cell growth factor. The activity present in HuT-102 supernatants was purified and initially termed IL-T (18, 19). In parallel, the corresponding factor was isolated from CV-1/EBNA cell culture supernatants and designated IL-15 (20). IL-15 is a 14–15 kDa glycoprotein encoded on human chromosome 4q31 and is expressed across a wide range of cell types. (21). Structurally, IL-15 is a class I helical cytokine with a four-*α*-helix bundle fold in which the helices are arranged in the characteristic up-up-down-down topology and is a member of the class I four-helix bundle cytokine family, which includes γc-dependent cytokines such as IL-2, IL-4, IL-7, and IL-9 (22). Physiologically, IL-15 is co-expressed with its high-affinity receptor IL-15R*α* and is produced in limited amounts as a soluble cytokine. Instead, IL-15 is predominantly delivered through trans-presentation by antigen-presenting cells (APCs), in which IL-15 is bound to IL-15R*α* on the surface of dentritic cells (DCs) and macrophages and presented to neighboring lymphocytes expressing the IL-2/IL-15R*β* and common *γ* chains (23). In this process, IL-15R*α* functions as a chaperone that stabilizes IL-15 intracellularly and traffics it to the plasma membrane for focused delivery to NK cells, NKT cells, and memory CD8^+^ T cells that express the shared IL-2R*βγ* signaling complex (24, 25) (**Figure 1**).

At the genetic level, IL-15 expression is regulated by a uniquely complex gene architecture that limits uncontrolled cytokine release. Although IL-15 mRNA is constitutively expressed across many cell types, including DCs, monocytes, macrophages, bone marrow stromal cells, and intestinal epithelial cells (26, 27), translation of IL-15 is tightly constrained by multiple regulatory elements, including upstream AUGs, alternative signal peptides, and inefficient secretion motifs. As a result, IL-15 protein is predominantly produced and displayed by monocytes, macrophages, and DCs, rather than being freely secreted. This multilayered post-transcriptional and intracellular control ensures that IL-15 activity is spatially restricted and delivered primarily through receptor-mediated trans-presentation rather than systemic diffusion.

IL-15 is tightly linked to innate immune activation and is upregulated in response to inflammatory and microbial stimuli. In APC, IL-15 expression increases following exposure to bacterial components such as lipopolysaccharide (LPS), type I IFN-*α/β*), type II IFN-*γ*, double-stranded RNA, and viral infection (28, 29). These signals promote coordinated induction of IL-15 and IL-15R*α*, enabling receptor-mediated trans-presentation to neighboring lymphocytes. This mode of IL-15 delivery is essential for the generation, maintenance, and functional specialization of IL-15-responsive immune populations, particularly NK cells and memory CD8^+^ T cells (23, 30, 31).

## 3 Divergent immunological effects of IL-2 and IL-15 signaling through the shared IL-2R*βγ* receptor

As we mentioned before, IL-2 and IL-15 are members of the common *γ*-chain ( γc) cytokine family, together with IL-4, IL-7, IL-9, and IL-21. Both cytokines transmit signals through a receptor complex consisting of IL-2 receptor *β* (IL-2R*β*, CD122) and the common *γ*-chain (CD132), thereby activating overlapping intracellular signaling cascades, including the JAK1/JAK3-STAT5, PI3K-AKT, and MAPK pathways. Early studies therefore proposed that IL-2 and IL-15 might exert largely overlapping biological functions. The distinct genomic, cellular, and functional characteristics of these two cytokines that differentiate their biological roles are summarized in **Table 1**. Subsequent clinical and experimental observations, however, have clearly demonstrated that engagement of this shared receptor complex can result in fundamentally different immune and therapeutic outcomes. These differences are particularly evident in clinical oncology. High-dose IL-2 therapy is capable of inducing durable tumor regression in a subset of patients with melanoma and renal cell carcinoma, but its broader application is constrained by severe systemic toxicity, preferential expansion of regulatory T cells, and induction of activation-induced cell death and functional exhaustion in effector T cells. In contrast, IL-15 preferentially sustains the survival, proliferation, and long-term maintenance of NK cells and memory CD8^+^ T cells, without driving regulatory T-cell expansion or terminal effector differentiation. These properties make IL-15 a more favorable cytokine for therapeutic strategies aimed at promoting durable antitumor immunity. The divergent biological effects of IL-2 and IL-15 do not arise from differences in their core IL-2R*βγ* signaling machinery, but instead reflect variations in receptor distribution, cytokine presentation, and context-dependent signal integration. A major determinant of this divergence is the differential utilization of cytokine-specific *α*-receptor chains. IL-2 binds with high affinity to IL-2R*α* (CD25), which is transiently expressed on activated T and B cells and constitutively expressed on regulatory T cells. In contrast, IL-15 associates with IL-15R*α*, a receptor that is predominantly expressed by activated monocytes and dendritic cells (9, 32).

**Table 1:** Comparative features of IL-2 and IL-15 biology and receptor engagement.

Property	IL-2	IL-15
Genomic organization	Composed of four exons and located on chromosome 4q26	Encoded by eight exons and mapped to chromosome 4q31
Primary cellular sources	Predominantly produced by activated CD4^+^ Th1 cells	Mainly synthesized by activated DCs and monocytes
Regulation of expression	Controlled at the transcriptional level and through mRNA stabilization	Largely regulated post-transcriptionally, including translational control and intracellular trafficking
Mode of receptor engagement	Signals primarily via cis-interaction with IL-2R*α*, IL-2/IL-15R*β*, and the common *γ*-chain co-expressed on activated T and B cells	Presented as an IL-15/IL-15R*α* complex on antigen-presenting cells and delivered in trans to NK cells and CD8^+^ memory T cells expressing IL-2/IL-15R*β* and the common *γ*-chain
Principal immunological role	Supports regulatory T-cell homeostasis and enforces peripheral tolerance through activation-induced cell death of autoreactive lymphocytes	Sustains NK cells and CD8^+^CD44*^hi^* memory T cells, enabling durable protective immune responses
Phenotype of cytokine or private receptor *α*-chain deficiency in mice	Severe lymphoproliferation with enlarged peripheral lymphoid organs and autoimmune manifestations due to uncontrolled T- and B-cell expansion	Profound loss of NK, NKT, *γ/δ* T cells, and CD8^+^CD44*^hi^* memory T-cell populations

Beyond differences in receptor expression, IL-2 and IL-15 also diverge in downstream signal integration. Although both cytokines activate the JAK1/JAK3-STAT5 axis, IL-15-induced T-cell proliferation relies strongly on FKBP12-mediated activation of p70 S6 kinase (p70S6K), whereas FKBP12 is dispensable for IL-2-driven proliferation (33). Consistent with this mechanism, FKBP12 deficiency selectively impairs ERK and p70S6K phosphorylation in response to IL-15 but not IL-2, indicating that IL-15 engages a distinct proliferative signaling module (33). Conversely, FKBP12.6 contributes to IL-2 signaling but not to IL-15, further reinforcing their mechanistic divergence (33). The most critical distinction between IL-2 and IL-15, however, lies in their modes of cytokine presentation. IL-2 is primarily secreted as a soluble cytokine or retained within the extracellular matrix, where it engages pre-assembled high-affinity IL-2R*αβγ* complexes on activated lymphocytes (24). This signaling configuration preferentially targets CD25-expressing cells, including regulatory T cells, thereby promoting immune regulation and terminal effector differentiation. In contrast, IL-15 is rarely released in a free form. Instead, it is co-expressed intracellularly with IL-15R*α* and retained on the surface of monocytes and dendritic cells. Inflammatory stimulation by type I or type II interferons, together with NF-*κ*B activation through CD40 or Toll-like receptor pathways, induces coordinated expression of IL-15 and IL-15R*α*, resulting in the formation of stable membrane-bound complexes that recycle through endosomal compartments and persist on the cell surface (24).

Within immunological synapses, IL-15 is delivered in trans by IL-15R*α* on antigen-presenting cells to neighboring lymphocytes that express IL-2R*βγ* but lack IL-15R*α* (34, 35). The principal targets of this trans-presentation mechanism are NK cells and memory CD8^+^ T cells, which express high levels of IL-2R*β* but minimal CD25. Although CD25 on dendritic cells can transiently present IL-2 in trans to antigen-specific T cells during early immune responses (24), this interaction does not reproduce the sustained, spatially restricted signaling characteristic of IL-15. In addition to trans-presentation, IL-15 can signal in cis on cells that co-express IL-15R*α*, IL-2R*β*, and the common *γ*-chain. This mode of signaling is particularly important for optimal NK-cell activation during inflammatory responses, as demonstrated in lipopolysaccharide-driven models (34). Moreover, IL-15 can engage non-canonical signaling pathways in certain innate immune cell types. In mast cells, IL-15 signals through alternative receptor complexes that recruit JAK2 and STAT5 rather than the classical JAK1/JAK3 pathways used in T cells (35). In myeloid cells, IL-15 can also transduce signals independently of IL-2R*βγ* and JAK/STAT through IL-15R*α*, JNK, and NF-*κ*B pathways to induce chemokine production, including RANTES (34).

Together, these receptor-level and signaling-context distinctions explain how IL-2 and IL-15, despite sharing IL-2R*βγ*, drive fundamentally different CD8^+^ T-cell fates. IL-2 signaling preferentially enforces regulatory and terminal effector programs, whereas IL-15 supports the persistence of long-lived, cytotoxic, and memory lymphocyte populations. IL-15-based cancer immunotherapies exploit this biology by delivering IL-15 through cis- or trans-presentation mechanisms that selectively engage IL-2R*βγ* on tumor-reactive lymphocytes, thereby promoting durable antitumor immunity while avoiding the immunosuppressive liabilities associated with IL-2.

Although IL-15 is best known for its critical role in the maintenance and activation of NK cells and memory CD8^+^ T cells, accumulating evidence indicates that IL-15 also influences several other immune cell populations relevant to tumor immunity. IL-15 can enhance the survival and functional activity of CD4^+^ T cells, particularly promoting the expansion of effector and memory subsets under inflammatory conditions. In addition, IL-15 has been shown to modulate dendritic cells, macrophages, and innate lymphoid cells, contributing to enhanced antigen presentation, cytokine production, and coordination of innate-adaptive immune responses. IL-15 signaling can also influence γδ T cells and NKT cells, which possess innate-like cytotoxic properties and are increasingly explored as platforms for cancer immunotherapy. Collectively, these findings highlight the broader immunomodulatory role of IL-15 across multiple immune compartments and further support its therapeutic potential in cancer immunotherapy.

## 4 IL-15 based cancer immunotherapy

### 4.1 rhIL-15

The clinical development of IL–15–based agents has progressed significantly, with several candidates currently being evaluated in human trials (**Table 2**). Central to these advancements, the clinical manufacture. The clinical manufacture of recombinant human interleukin-15 (rhIL-15) has been established using Escherichia coli (E. coli), providing a robust foundation for experimental and translational studies of IL-15-based immunotherapy (42). Owing to improvements in rhIL-15 purification and formulation, administration of rhIL-15 has been shown to induce tumor regression and suppress metastatic dissemination in murine cancer models, including the LA795 lung adenocarcinoma model. Tang and colleagues (43) demonstrated that rhIL-15 treatment resulted in a significantly lower tumor burden and prolonged survival compared with rhIL-2 in the LA795 transplantable lung adenocarcinoma model, indicating a superior anti-tumor efficacy of rhIL-15 at equivalent doses. In addition, IL-15-mediated anti-tumor activity has been widely reported across other transplantable tumor systems, including melanoma, colon carcinoma, carcinoma-derived lines, and lymphomas in preclinical studies (44).

**Table 2:** Recent clinical advances in IL-15–based cancer immunotherapy.

Agent/strategy	Indication	Clinical stage	Key findings	Ref.
Anktiva (N-803)	NMIBC	FDA Approved	High complete response; durable remissions	(36)
N-803 post-HCT	AML/MDS	Phase I/II	NK expansion; antitumor surveillance	(37)
NKTR-255 + CAR-T	B-ALL/LBCL	Phase I	Enhanced immune expansion; early efficacy	(38)
NIZ985 (IL-15/IL-15R*α*)	Solid tumors	Phase I	Cytokine induction; lymphocyte proliferation	(39)
SOT201 (PD-1–IL-15 mutein)	Solid tumors	Phase I	Selective stimulation of PD-1^+^ TILs	(40)
PF-07209960 (anti–PD-1–IL-15m)	Solid tumors	Phase I	PK/PD and safety reported	(41)

In addition to its therapeutic efficacy, the safety of rhIL-15 has been evaluated in multiple preclinical and clinical studies. Preclinical investigations demonstrated that rhIL-15 exhibits an acceptable safety profile across several dosing regimens. Administration of rhIL-15 in non-human primates by continuous intravenous infusion at 20 *µ*g/kg/day for 10 days was well tolerated, with no lasting effects on clinical status, body weight, or food consumption. At higher doses (40 *µ*g/kg/day), treatment resulted in dose-dependent but transient laboratory abnormalities, including reversible elevations in liver enzymes and occasional neutropenia, all of which resolved following treatment discontinuation. Importantly, no progressive or irreversible toxicities were observed, supporting the overall tolerability of short-term IL-15 administration (45). In this study, IL-15 administration also induced marked expansion of circulating NK cells and effector memory CD8^+^ T cells, highlighting its potential to promote durable anti-tumor immunity (45). Consistent with its physiological role in lymphocyte homeostasis, IL-15 preferentially expands NK cells and effector-memory CD8^+^ T cells *in vivo*, as demonstrated in rhesus macaques (46). Building on these encouraging preclinical findings, a phase I dose-escalation trial evaluated subcutaneous rhIL-15 in patients with advanced refractory solid tumors, including melanoma, renal cell carcinoma, non-small cell lung cancer, and head and neck cancer (47) (ClinicalTrials.gov identifier: NCT01727076). In this clinical trial, rhIL-15 was administered as daily injections for two weeks of a four-week cycle, with dose levels ranging from 0.25 to 3.0 µg /kg/day. The primary objectives were to assess safety, tolerability, and the maximum tolerated dose, as well as pharmacodynamic immune responses and preliminary clinical activity. rhIL-15 was generally well tolerated; however, dose-limiting toxicities at higher dose levels included pancreatitis and transient cardiovascular effects, establishing 2 *µ*g/kg/day as the maximum tolerated dose. Although no objective tumor regressions were observed, several patients achieved disease stabilization, supporting continued development of IL-15-based therapies, particularly in combination with other immunotherapeutic strategies.

Despite its promising biological activity, rhIL-15 monotherapy faces several important limitations. Effective expansion and maintenance of immune effector cells require sustained exposure to IL-15 at biologically active concentrations (48). However, maintaining durable levels of soluble IL-15 (sIL-15) *in vivo* is challenging due to its short serum half-life, reported to be approximately 2–3 hours following intravenous administration, together with relatively rapid systemic clearance (5–11 L/h depending on dose) (47, 48). A key determinant of IL-15 stability and bioavailability is its high-affinity receptor IL-15R*α*, which governs IL-15 trafficking, presentation, and persistence in circulation (23). Collectively, these constraints limit the therapeutic efficacy of unmodified IL-15 and have driven the development of engineered IL-15 derivatives designed to improve pharmacokinetics, receptor engagement, and targeted delivery.

### 4.2 IL-15 modification and IL-15/IL-15R*α* complex

To address the limitations associated with rhIL-15, two major optimization strategies have been explored. The first approach involves engineering IL-15 itself through mutations or other molecular modifications, a strategy commonly used to improve the pharmacological properties of protein therapeutics. The second strategy focuses on the development of IL-15/IL-15R*α* complexes. Because IL-15 naturally functions as a heterodimeric IL-15-IL-15R*α* complex *in vivo* (hetIL-15), researchers hypothesized that mimicking this physiological form could enhance cytokine stability and biological activity compared with IL-15 monomers. Subsequent studies have supported this concept, demonstrating improved pharmacokinetic and immunostimulatory properties for IL-15/IL-15R*α*-based agonists. Notably, nogapendekin alfa inbakicept (N-803) is currently the first and only IL-15-based immunotherapy approved by the U.S. FDA, representing a major milestone in the clinical translation of IL-15 agonists.

N-803 was named after being purchased by ImmunoBio after the name of ALT-803 which is a 2 (IL-15/IL-15R*α*) tetramer formed by the assembly of two IL-15 mutants (IL-15 N72D, which has a higher affinity for the receptor) and two IL15R*α* Su-IgG1 Fc fusion proteins (49). The combination of N-803 with Bacillus Calmette-Guérin (BCG) was approved by the FDA for patients with BCG-unresponsive non-muscle invasive bladder cancer (NMIBC) (36). This approval was supported by the results of the multicenter trial (NCT0302285), which evaluated intravesical N-803 together with BCG in patients with high-risk NMIBC with carcinoma *in situ* with or without papillary tumors (50). In this study, 62% of the 77 enrolled patients achieved a complete response, and 58% of these responses were sustained for at least 12 months, exceeding the benchmark complete response rate of 30% at 1 year proposed by FDA experts and the International Bladder Cancer Group (51–53). The development of this therapeutic strategy was motivated by earlier preclinical studies aiming to enhance BCG-mediated antitumor immunity. Although intravesical BCG can induce durable responses in NMIBC, approximately 40% of patients experience disease recurrence and become unresponsive to further BCG therapy (54–56). Preclinical bladder cancer models demonstrated that the combination of IL-15 and intravesical BCG administration was safe and significantly enhanced antitumor immune responses (57–59). While the precise mechanisms were not fully elucidated, improved outcomes were associated with the expansion of NK cells and increased production of inflammatory cytokines. Collectively, these findings provided the rationale for clinical evaluation of the N-803 and BCG combination in NMIBC patients.

In addition, FIST15 which links the IL-15R*α* sushi domain-IL-15 complex to tandem TGF-*β* traps derived from TGF-*β*RII-ECD, induces potent NK cell-dependent antitumor activity in syngeneic mouse models (63). Building on this concept, HCW9218 was generated using a soluble tissue factor-based scaffold to integrate the IL-15R*α*-IL-15 complex with TGF-*β*RII-ECD. This bifunctional molecule promoted intratumoral accumulation of NK cells and CD8^+^ T cells and induced strong antitumor responses in preclinical models (60). In early clinical testing, HCW9218 was well tolerated and induced marked NK-cell expansion following a single dose, with dose escalation ongoing up to 0.5 mg/kg (NCT05322408). Beyond IL-15 mutations and IL-15/IL-15R*α* complex engineering, another important strategy to enhance the therapeutic potential of IL-15 involves the development of IL-15-based immunocytokines.

### 4.3 IL-15-based immunocytokines

**Figure 2:**
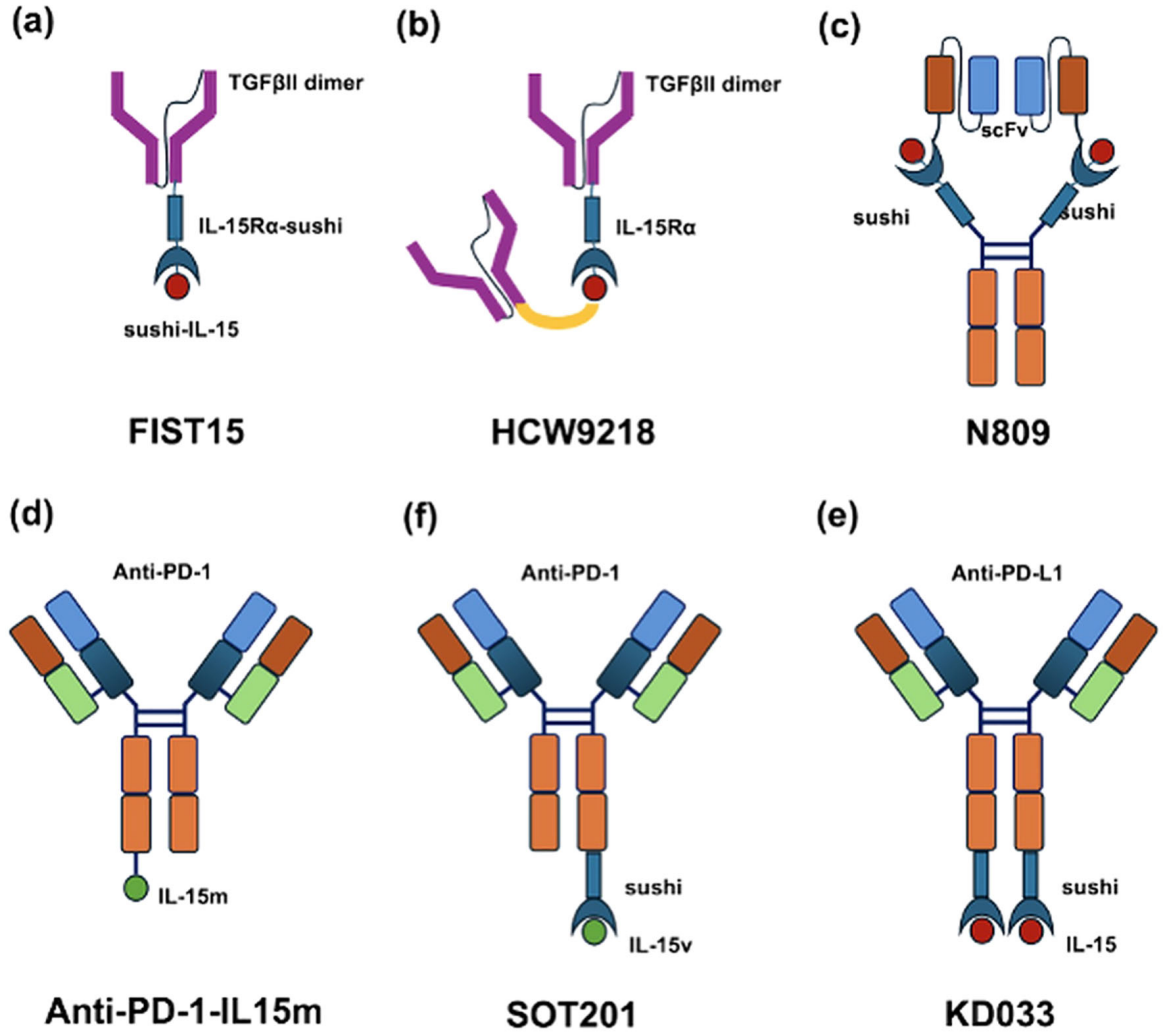
Structural designs of IL-15-based immunocytokines for cancer immunotherapy. **(a)** FIST15, a bifunctional fusion protein consisting of a TGF-*β* receptor II (TGF*β*RII) ectodomain dimer fused to an IL-15/IL-15R*α* sushi complex, designed to simultaneously neutralize TGF-*β* and activate IL-15 signaling (63). **(b)** HCW9218, an optimized TGF*β*RII-IL-15/IL-15R*α* fusion protein enhancing NK and CD8^+^ T cell activation while blocking TGF-*β* signaling (64). **(c)** N-809, an IL-15 superagonist complex fused to anti-PD-L1 single-chain variable fragments (scFv) to direct IL-15 signaling to PD-L1^+^ tumors (65). **(d)** Anti-PD-1-IL-15m, an engineered IL-15 mutein fused to an anti-PD-1 antibody to selectively stimulate PD-1^+^ tumor-infiltrating T cells (66). **(e)** KD033, a fully human anti-PD-L1 antibody fused to an IL-15/IL-15R*α* sushi complex for tumor-targeted IL-15 delivery (67). **(f)** SOT201, an Fc silenced anti-PD-1 antibody fused to a cis-acting IL-15/IL-15R*βγ* selective sushi mutein.

Antibody-cytokine fusion proteins, commonly referred to as immunocytokines, represent a targeted strategy for cytokine delivery and have been extensively developed and evaluated in clinical studies across a broad range of oncologic and inflammatory indications. These engineered molecules typically comprise a targeting antibody domain linked via a flexible peptide linker to a cytokine effector. By concentrating cytokine activity at disease sites, immunocytokines can reduce systemic exposure and toxicity associated with conventional cytokine therapy, thereby expanding the therapeutic window of the cytokine payload (61, 62). To enhance and spatially restrict IL-15-mediated immune activation, multiple IL-15-based immunocytokines have been engineered, as illustrated in **Figure 2**, including fusion constructs targeting immune checkpoint molecules such as PD-1 and PD-L1, as well as immunomodulatory proteins such as the transforming growth factor-*β* receptor II ectodomain (TGF-*β*RII-ECD). Several of these molecules are currently in preclinical and early-stage clinical development and have demonstrated robust immune-stimulating activity in murine tumor models, primarily through expansion and functional enhancement of NK cells and CD8^+^ T cells.

In addition to enabling tumor-targeted cytokine delivery, antibody-based immunocytokines substantially improve the pharmacokinetic properties of IL-15. In contrast to soluble rhIL-15, IL-15/IL-15R*α* complexes and Fc-containing immunocytokines demonstrate markedly prolonged systemic persistence due to receptor stabilization and FcRn-mediated recycling. Preclinical and clinical studies have reported circulatory half-lives ranging from approximately 20–30 hours for IL-15/IL-15R*α* superagonist complexes to several days for antibody-based IL-15 immunocytokines, resulting in improved bioavailability and sustained activation of NK cells and CD8^+^ T cells. These pharmacokinetic improvements provide a key rationale for the development of targeted IL-15 immunocytokine platforms.

Several next-generation IL-15 immunocytokines have further integrated cytokine signaling with immune checkpoint blockade to enhance tumor-selective immune activation. N-809 is a bifunctional fusion protein composed of an IL-15/IL-15R*α* superagonist linked to an IgG1 Fc domain bearing two anti-PD-L1 single-chain variable fragments. In murine models of breast and colon carcinoma, N-809 demonstrated superior antitumor activity compared with the combination of non-targeted IL-15 and anti-PD-L1. This efficacy was associated with enhanced activation and recruitment of NK cells and CD8^+^ T cells within tumor-draining lymph nodes and the tumor microenvironment, as well as chemokine-mediated effector cell trafficking into tumors (64, 68). An alternative approach was developed by Pfizer with anti-PD-1-IL-15m, an immunocytokine in which a PD-1-specific antibody is fused to an IL-15 mutein lacking IL-15R*α* binding and exhibiting reduced affinity for IL-2R*βγ*. This design reduced receptor-mediated clearance and prolonged systemic exposure relative to wild-type anti-PD-1-IL-15, resulting in improved pharmacokinetic persistence and sustained immune activation *in vivo*. In preclinical studies, a single subcutaneous dose of anti-PD-1-IL-15m elicited strong, dose-dependent antitumor responses that exceeded those achieved with combined IL-15 superagonist and anti-PD-1 therapy. However, dosing at 5 mg/kg was associated with body-weight loss driven by peripheral NK-cell expansion. Mechanistically, tumor control depended primarily on CD8^+^ T cells rather than NK cells, with selective expansion of a proliferative, effector-like exhausted CD8^+^ T-cell population within tumors (66). Another PD-1-targeted immunocytokine, SOT201, consists of an Fc-silenced humanized anti-PD-1 antibody fused to a covalent sushi/IL-15 mutein with reduced IL-2R*βγ* affinity. This construct selectively stimulates PD-1^+^ tumor-infiltrating lymphocytes, restoring exhausted T-cell function *in vitro* and expanding antigen-specific PD-1^+^ CD8^+^ T cells *in vivo*. SOT201 demonstrated potent antitumor activity in both PD-1-sensitive and PD-1-resistant tumor models and exhibited favorable pharmacokinetics with prolonged systemic persistence in non-human primates, supporting its ongoing evaluation in a phase I clinical trial (NCT06163391) (69).

In addition to PD-1-targeted designs, KD033 is a homodimeric immunocytokine composed of a fully human anti-PD-L1 antibody fused to the IL-15R*α* sushi domain-IL-15 complex. In syngeneic tumor models, a single dose of KD033 induced durable antitumor responses with a favorable safety profile, reflecting the extended systemic persistence and improved bioavailability conferred by the IL-15/IL-15R*α* complex and antibody scaffold. (67). Early clinical evaluation further demonstrated that KD033 was generally well tolerated, with manageable adverse events.

### 4.4 IL-15 in adaptive cell therapy

Genetic modification has become central to the development of adoptive cell therapies for cancer, enabling precise control over immune cell survival, function, and persistence. Current engineering approaches include the introduction of cytokine or chemokine support, modulation of transcriptional programs, disruption of inhibitory immune checkpoints, and incorporation of inducible safety switches (70). Among these strategies, the ectopic expression of human IL-15 has emerged as a particularly powerful method to enhance the therapeutic durability and antitumor activity of engineered immune cells, as summarized in the chronological overview of clinical trials involving IL–15–armored cellular therapies (**Table 3**).

**Table 3:** Chronologically ordered clinical trials of IL-15-armored cellular therapies in cancer immunotherapy.

Start year	Therapy	Malignancies	Description (cell type, target, IL-15 design)	Clinical trial
2015	CD19 CAR-T (IL-15 armored)	Recurrent DLBCL; recurrent FL; recurrent MCL	Autologous CD19-targeted CAR-T cells engineered to express IL-15 to enhance T cell persistence and antitumor activity; IL-15 construct details not publicly disclosed	NCT02652910
2017	CD19 CAR-T (IL-15 armored)	B-lymphoid malignancies; ALL; CLL; NHL	Autologous CD19-directed CAR-T cells with IL-15 co-expression to promote T-cell expansion and survival; IL-15 expression cassette reported but promoter and variant not specified	NCT03056339
2019	CD19 CAR-T (IL-15 armored)	MCL; relapsed/refractory DLBCL; FL; B-cell NHL	Autologous CD19 CAR-T cells engineered with IL-15 signaling support to enhance proliferation and persistence; IL-15 construct design not disclosed	NCT03579927
2019	GD2 CAR-T (IL-15 armored)	Neuroblastoma; osteosarcoma	GD2-targeting CAR-T cells engineered to express IL-15 to improve persistence and antitumor immune activity; IL-15 variant and promoter not reported	NCT03721068
2020	CD19 CAR-T (IL-15 armored)	Refractory B-NHL; SLL; relapsed ALL; relapsed CLL; relapsed NHL	CD19-directed CAR-T cells with IL-15 co-expression to enhance immune expansion and persistence; IL-15 expression cassette details not publicly available	NCT03774654
2020	GPC3 CAR-T (IL-15 armored)	Hepatocellular carcinoma; hepatoblastoma	GPC3-targeting CAR-T cells engineered to express IL-15 to improve antitumor activity in solid tumors; IL-15 construct details not reported	NCT04093648
2021	CD19 CAR-T (IL-15 armored)	ALL; B-cell lymphoma; CLL	CD19-targeted CAR-T cells engineered with IL-15 expression to support proliferation and survival of transferred T cells; IL-15 promoter and variant not specified	NCT04814004
2021	GPC3 CAR-T (IL-15 armored)	Liver cancer; RMS; malignant rhabdoid tumor; liposarcoma; Wilms tumor; yolk sac tumor	GPC3-directed CAR-T cells expressing IL-15 to enhance persistence and tumor infiltration; detailed IL-15 construct design not disclosed	NCT04377932
2021	GPC3 CAR-T (IL-15 armored)	Liver cancer; RMS; malignant rhabdoid tumor; liposarcoma; tumor; Wilms tumor; yolk sac tumor	GPC3 CAR-T cells with IL-15 expression module designed to enhance immune activation and antitumor responses; IL-15 construct details not reported	NCT05103631
2022	CD70 CAR-T (IL-15 armored)	B-cell lymphoma; MDS; AML	CD70-targeting CAR-T cells engineered to express IL-15 to enhance immune expansion and persistence; IL-15 expression system details not disclosed	NCT05092451
2022	PD-L1 armored T cells (IL-15 engineered)	Advanced/metastatic/refractory NSCLC	Engineered T cells targeting PD-L1 with IL15 expression module to enhance immune activation and persistence; IL-15 construct details not publicly available	NCT05334329
2024	CD5 CAR-T (IL-15 armored)	T-cell malignancies; mantle cell lymphoma; CLL	CD5-directed CAR-T cells engineered with IL-15 expression to support proliferation and antitumor activity; IL-15 promoter and variant not disclosed	NCT05110742
2024	GPC3 CAR-T (IL-15 armored)	Liver cancer; RMS; malignant rhabdoid tumor; liposarcoma; Wilms tumor; yolk sac tumor	GPC3-targeted CAR-T cells expressing IL- 15 to enhance persistence and tumor killing; IL-15 construct details not publicly disclosed	NCT04715191

The table summarizes the engineered cell type, targeted tumor-associated antigen (TAA), and available information regarding IL-15 expression constructs where reported. Abbreviations: IL-15, interleukin-15; DLBCL, diffuse large B-cell lymphoma; FL, follicular lymphoma; MCL, mantle cell lymphoma; ALL, acute lymphoblastic leukemia; CLL, chronic lymphocytic leukemia; NHL, non-Hodgkin lymphoma; RMS, rhabdomyosarcoma; MDS, myelodysplastic syndrome; AML, acute myeloid leukemia; NSCLC, non-small cell lung cancer.

In contrast to soluble cytokine therapies, the persistence of IL-15-engineered cellular therapies is not primarily determined by cytokine pharmacokinetics but rather by the *in vivo* expansion and survival of the engineered immune cells. Clinical and preclinical studies have shown that CAR-T and CAR-NK cells expressing IL-15 can persist in circulation for several weeks to months, reflecting sustained autocrine or membrane-associated IL-15 signaling that promotes long-term cell survival and functional competence (71). This prolonged cellular persistence represents a key advantage of IL-15-armored adoptive cell therapies compared with systemic cytokine administration.

IL-15 has been incorporated into a wide range of CAR-based cellular platforms, including conventional T cells (71–73), natural killer (NK) cells (74, 75), invariant NKT cells (76–79), *γδ* T cells (80), and macrophages (81). Across these diverse immune lineages, IL-15 expression consistently promotes improved cell survival, sustained proliferation, and enhanced cytotoxic function *in vivo*, thereby addressing key limitations of conventional adoptive cell therapies.

#### 4.4.1 IL-15-armored CAR T cells

To enhance the persistence, expansion, and antitumor efficacy of CAR-T cells, multiple groups have explored genetic strategies to incorporate IL-15 signaling directly into CAR-T cell products. Early preclinical studies demonstrated that constitutive or regulated IL-15 expression could overcome key limitations of conventional CAR-T cells, including activation-induced cell death, functional exhaustion, and insufficient *in vivo* persistence. Hoyos and colleagues first reported a CD19-targeted CAR-T construct co-expressing IL-15 together with an inducible caspase-9 (iC9) suicide gene (iC9/CAR.19/IL-15), which significantly enhanced CAR-T expansion, survival, and antitumor activity in preclinical models while retaining a pharmacologic safety mechanism to eliminate the cells in the event of toxicity (73). Building on this concept, Hurton et al. developed CAR-T cells expressing a membrane-bound IL-15 (mbIL-15) fusion protein, enabling sustained autocrine and juxtacrine IL-15 signaling that promoted long-term persistence and memory-like phenotypes without excessive systemic cytokine release (82). Similar strategies were subsequently applied to solid tumor targets, including IL13R*α*-CAR (83) and Fn14-CAR (84) constructs for glioblastoma, where IL-15 armoring enhanced intratumoral persistence and antitumor efficacy in otherwise highly immunosuppressive environments.

Encouraged by these preclinical findings, IL-15-armed CAR-T cells have progressed into early clinical evaluation. In 2021, IL-15-expressing CAR-T cells were administered to patients with relapsed or refractory B-cell acute lymphoblastic leukemia (B-ALL) following failure of both CD19- and CD22-directed CAR-T therapies Sun et al. (85), demonstrating feasibility and clinical activity in a heavily pretreated population. A phase I study published the same year further supported the safety of IL-15 incorporation, showing enhanced CAR-T expansion and persistence with manageable toxicity profiles (85). Collectively, these early clinical experiences suggested that incorporating IL-15 and its receptor complex represents a feasible and potentially effective strategy to potentiate CAR-T cell therapy.

More recent preclinical work has extended IL-15 armoring strategies to the tumor stroma. FAP-targeted CAR-T cells engineered to secrete IL-15 demonstrated robust antitumor activity in solid tumor models by enhancing CAR-T survival, promoting memory-like differentiation, and reshaping the tumor microenvironment, highlighting the versatility of IL-15-based CAR-T engineering beyond direct tumor cell targeting (86). Importantly, definitive clinical and translational evidence for the utility of IL-15-armored CAR-T cells in solid tumors was recently provided by a study reporting outcomes from patients treated with GPC3-targeted CAR-T cells co-expressing IL-15 (71). Compared with conventional GPC3 CAR-T cells, IL-15-armored CAR-T cells exhibited markedly enhanced *in vivo* expansion, persistence, and tumor infiltration, translating into improved disease control in a subset of patients with advanced solid tumors. Although IL-15 co-expression increased the incidence of cytokine-related toxicities, these adverse events were manageable using standard interventions and activation of the inducible caspase-9 safety switch. Notably, integrated correlative analyses revealed distinct transcriptional programs in tumor-infiltrating CAR-T cells associated with clinical response, providing mechanistic insight into how IL-15 enhances CAR-T functional fitness within the hostile solid tumor microenvironment.

Collectively, these studies establish IL-15 armoring as a powerful strategy to enhance CAR-T cell persistence and efficacy across both hematologic malignancies and solid tumors, while underscoring the importance of incorporating safety mechanisms to balance potency and tolerability.

#### 4.4.2 IL-15-armed CAR-NK cell therapy

NK cells represent an attractive platform for adoptive cell therapy due to their intrinsic cytotoxicity, lack of graft-versus-host disease risk, and suitability for allogeneic “off-the-shelf” use (87). However, the clinical application of CAR-NK cells has historically been limited by insufficient *in vivo* persistence and expansion (74). Given the central role of IL-15 in NK cells development, survival, and activation, genetic incorporation of IL-15 into CAR-NK cells has emerged as a rational strategy to enhance their therapeutic potency. Preclinical studies demonstrated that CAR-NK cells engineered to express IL-15 or IL-15-based receptor complexes exhibit markedly improved proliferation, metabolic fitness, and resistance to apoptosis, leading to enhanced antitumor activity across hematologic and solid tumor models.

In contrast to systemic cytokine administration, autocrine or membrane-associated IL-15 delivery enables sustained NK-cell support while minimizing systemic exposure and toxicity. These findings were translated into the clinic with the development of allogeneic CD19-specific CAR-NK cells derived from umbilical cord blood and engineered to express IL-15. Initial first-in-human results reported in 2020 established the feasibility and safety of this approach (74), demonstrating rapid tumor responses in patients with relapsed or refractory CD19-positive lymphoid malignancies without cytokine release syndrome, neurotoxicity, or graft-versus-host disease. Importantly, IL-15 expression supported robust *in vivo* expansion and persistence of CAR-NK cells for several weeks to months, exceeding the typically short persistence of unmodified NK-cell therapies.

Subsequent long-term follow-up and expanded cohort analyses from the same phase 1/2 clinical trial provided a more comprehensive assessment of efficacy, durability, and biological determinants of response. Mature data published in 2024 confirmed durable clinical remissions in a subset of patients and identified immune correlates associated with treatment response, including CAR-NK cell persistence, activation status, and transcriptional programs linked to cytotoxic function Marin et al. (88). These analyses demonstrated that IL-15 armoring not only enhances early expansion but also sustains functional competence of CAR-NK cells over time, addressing a central limitation of earlier NK-cell-based therapies. Beyond hematologic malignancies, IL-15-armed CAR-NK strategies are being actively explored for solid tumors. Preclinical studies have shown that IL-15 expression improves CAR-NK infiltration, survival, and antitumor efficacy within immunosuppressive tumor microenvironments, supporting broader application of this platform (89). Collectively, these findings position IL-15-armored CAR-NK cells as a promising next-generation cellular immunotherapy that combines the safety advantages of NK cells with enhanced persistence and potency conferred by IL-15 signaling.

#### 4.4.3 IL-15 armed unconventional CAR T cells

Beyond conventional αβ T cells and NK cells, IL-15 has also been incorporated into adoptive immunotherapies based on unconventional T-cell subsets, including γδ T cells and invariant natural killer T (iNKT) cells. Unlike classical T cells that rely on major histocompatibility complex (MHC) restricted antigen recognition, these unconventional T cells recognize non-polymorphic ligands, thereby minimizing the risk of graft-versus-host disease (GvHD) and supporting their use as allogeneic, off-the-shelf cellular therapies.

Early preclinical work demonstrated the feasibility of this strategy in *γδ* T cells. Makkouk et al. engineered γδ T cells with a glypican-3 (GPC3)-specific CAR incorporating a 4-1BB/CD3  ζ signaling domain together with IL-15 (90). In xenograft models of hepatocellular carcinoma, IL-15-armored CAR γδ T cells exhibited superior proliferation, persistence, and antitumor activity compared with CAR γδ T cells lacking IL-15, underscoring the ability of IL-15 to enhance functional fitness in this unconventional T-cell platform. Similar IL-15-based engineering approaches have been extended to iNKT cells, which combine innate-like cytotoxicity with favorable tumor-homing properties. Xu and colleagues developed a GD2-targeted CAR optimized for iNKT cells that co-expresses IL-15, enabling enhanced expansion and survival of the engineered cells (76). This strategy advanced into early-phase clinical testing in children with relapsed or refractory neuroblastoma (ClinicalTrials.gov identifier: NCT03294954) (76). Initial clinical observations indicated that IL-15-armored GD2-CAR iNKT cells expanded *in vivo*, trafficked to tumor sites, and were well tolerated, supporting the feasibility of this approach.

Recent clinical advances have highlighted the therapeutic potential of invariant natural killer T (iNKT) cells engineered with chimeric antigen receptors (CARs) for the treatment of high-risk neuroblastoma. In the first-in-human phase 1 study evaluating autologous GD2-specific CAR iNKT cells co-expressing interleukin-15 (GD2-CAR.15), treatment was well tolerated, with no dose-limiting toxicities and only a single, manageable episode of grade 2 cytokine release syndrome (91). Collectively, these emerging clinical and translational findings reinforce the concept that IL-15 armoring can substantially improve the efficacy of γδT- and iNKT-cell-based therapies, broadening the scope of IL-15-enabled adoptive cell platforms beyond conventional CAR-T and CAR-NK approaches.

### 4.5 IL15-based combination immunotherapy strategies

Several interventions combining IL-15 agents with checkpoint inhibitors, antibodies stimulating immune cell functions (e.g., anti-CD40, anti-CD16), monoclonal antibodies specific for tumor-associated antigens, and immunotoxins have been tested in preclinical models, with promising results.

#### 4.5.1 Combination of IL-15 with checkpoint inhibitors

IL-15-mediated immune activation can also induce compensatory immunoregulatory mechanisms that may limit sustained antitumor responses. For instance, IL-15 stimulation has been associated with the upregulation of inhibitory receptors such as T-cell immunoglobulin and mucin domain-3 (TIM-3) and T-cell immunoreceptor with Ig and ITIM domains (TIGIT) on CD8 ^+^ T cells, accompanied by increased secretion of the anti-inflammatory cytokine IL-10, which may attenuate effector immune activity within the tumor microenvironment (92, 93). These observations provide a strong rationale for combining IL-15-based therapies with immune checkpoint blockade in order to counteract adaptive immune resistance and sustain cytotoxic lymphocyte function.

Preclinical studies have demonstrated encouraging outcomes for such combination approaches. In murine tumor models, subcutaneous administration of rhIL-15 together with antibodies targeting CTLA-4 and PD-L1 was evaluated in CT26 and MC38 colon carcinoma models as well as in the Transgenic Adenocarcinoma of Mouse Prostate C2 (TRAMP-C2) model. Although treatment with each individual agent produced only limited therapeutic activity, the triple combination therapy resulted in a marked enhancement of antitumor efficacy, highlighting the synergistic potential of integrating IL-15-driven immune activation with checkpoint inhibition (92).

Similar findings have been reported with IL-15 superagonists. The IL-15/IL-15R*α* superagonist complex N-803 has shown significant synergy when combined with checkpoint inhibitors, promoting the development of durable immune responses and further enhancing the antitumor effects of the individual treatments (94, 95). Likewise, the IL-15 receptor ligand fusion protein RLI has been demonstrated to potentiate the therapeutic efficacy of PD-1 blockade in preclinical tumor models (96). In addition to the well-established PD-1/PD-L1 and CTLA-4 pathways, emerging inhibitory receptors are also being investigated as potential combinatorial targets. Among these, TIGIT has recently gained attention as a promising checkpoint molecule for integration with IL-15-based immunotherapeutic strategies (97).

#### 4.5.2 Combination of IL-15 with antibodies

In addition to checkpoint blockade, IL-15-based therapies have also been investigated in combination with agents that enhance antigen presentation and immune cell activation. One example involves the use of agonistic anti-CD40 antibodies, which can restore CD4^+^ T-cell helper activity and thereby promote the generation of tumor-specific CD8^+^ T cells. In the TRAMP-C2 prostate cancer model, co-administration of IL-15 with anti-CD40 antibodies resulted in a substantial expansion of TRAMP-C2-specific CD8^+^ T cells approximately a ten-fold increase, leading to improved tumor growth control compared with either treatment alone (98–100). These findings highlight the potential of combining IL-15-driven lymphocyte activation with strategies that enhance T-cell priming and help.

Another major application of IL-15 combinations exploits its potent stimulatory effects on NK cells. Because NK cells mediate antibody-dependent cellular cytotoxicity (ADCC), IL-15 has been extensively evaluated alongside therapeutic monoclonal antibodies to amplify antitumor responses. Preclinical studies demonstrated that recombinant human IL-15 (sch rhIL-15) enhances the efficacy of several clinically relevant antibodies, including cetuximab in breast cancer models (101, 102), rituximab in chronic lymphocytic leukemia and CD20-expressing lymphoma models (103, 104), and alemtuzumab in xenograft models of adult T-cell leukemia/lymphoma (103). These combinations improve NK-cell activation and significantly increase ADCC-mediated tumor cell killing.

IL-15 superagonists have also been integrated into antibody-based immunotherapy strategies. The IL-15 superagonist complex N-803 has shown strong synergy with anti-CD20 monoclonal antibodies by enhancing NK-cell activation and ADCC against B-cell lymphomas (105, 106). Building on this concept, engineered antibody platforms such as bispecific and trispecific killer engagers (BiKEs and TriKEs) have been developed to further potentiate NK-cell responses. These molecules form an immunological bridge between NK cells and tumor cells by incorporating single-chain variable fragments (scFvs) targeting the CD16 activating receptor on NK cells and tumor-associated antigens such as CD19, CD20, or CD33, while simultaneously delivering IL-15 to support NK-cell survival and expansion *in vivo* (107–109).

More recently, fusion proteins incorporating IL-15 agonists have been designed to enhance both NK- and T-cell-mediated antitumor activity. For example, a fusion construct combining the IL-15 receptor ligand (RLI) with an anti-GD2 antibody targeting the tumor-associated disialoganglioside GD2 demonstrated potent antitumor activity in EL4 and metastatic NXS2 mouse models, primarily through enhanced ADCC (110). In addition, multifunctional fusion proteins integrating tumor-specific antibodies, RLI, and co-stimulatory ligands such as 4-1BBL, OX40L, or glucocorticoid-induced TNF receptor ligand (GITRL) have shown the ability to reduce lung metastasis in the B16-FAP melanoma model. These constructs promoted T-cell proliferation, increased cytotoxic activity, and stimulated interferon- production, further illustrating the versatility of IL-15-based combination platforms in cancer immunotherapy (111, 112).

#### 4.5.2 Combination of IL-15 with *γ*-chain family of cytokines

As mentioned before, members of the common *γ*-chain cytokine family, including IL-2, IL-7, IL-15, and IL-21, share receptor signaling pathways and collectively regulate lymphocyte proliferation, survival, and differentiation. Increasing evidence suggests that combining IL-15 with other *γ*c cytokines may enhance immune activation and antitumor responses. For example, IL-7 and IL-15 have complementary roles in maintaining lymphocyte homeostasis and promoting immune recovery (113). In addition, *γ*-chain cytokines cooperate in regulating peripheral T-cell expansion and survival (114). Experimental studies further indicate that dual cytokine strategies can enhance antitumor immunity. For instance, stimulation of lymphocytes from breast cancer patients with IL-7 and IL-15 significantly increased lymphocyte proliferation and viability compared with single cytokine treatment (115). Similarly, a recombinant IL-7/IL-15 fusion cytokine demonstrated enhanced antitumor activity in murine melanoma and colon cancer models, which was associated with increased infiltration of T cells, dendritic cells, and NK cells, as well as reduced regulatory T-cell populations (116). These findings suggest that combining IL-15 with other *γ*c cytokines may represent a promising strategy to improve antitumor immune responses.

## 5 Future directions

IL-15 has emerged as a cornerstone cytokine for next-generation cancer immunotherapy because of its unique capacity to expand and sustain NK cells and CD8^+^ T cells without promoting regulatory T-cell dominance or activation-induced cell death. Accumulating preclinical and clinical evidence supports the therapeutic potential of IL-15 to drive durable antitumor immunity (93). However, IL-15 signaling is embedded within a highly interconnected cytokine network, and increasing evidence suggests that certain IL-15 isoforms or receptor complexes may also contribute to immune dysregulation and tumor progression under pathological conditions (117). Elevated levels of soluble IL-15 or IL-15R*α* in cancer patients correlate with adverse outcomes, likely reflecting chronic inflammatory signaling rather than a direct oncogenic effect (118). These findings highlight the need to define context-dependent IL-15 signaling states using genetic and systems-level approaches.

One major translational challenge is controlling the intensity and distribution of IL-15 signaling. Although IL-15 superagonists such as N-803 exhibit enhanced stability and biological activity, their extended half-life requires high clinical doses that can result in dose-limiting toxicities (119). Ongoing clinical trials of IL-15 derivatives will be critical for identifying therapeutic windows that maximize immune activation while limiting systemic inflammation. In addition to dosing and pharmacokinetic constraints, IL-15-driven immune activation is also regulated by inhibitory checkpoint pathways within the tumor microenvironment. Emerging evidence suggests that IL-15 stimulation can induce the upregulation of immune checkpoint molecules such as PD-1 on activated CD8^+^ T cells and PD-L1 on tumor or myeloid cells. This feedback mechanism may function as a physiological brake that prevents excessive immune activation but may also limit the efficacy of IL-15 monotherapy. Consistent with this concept, previous studies have reported increased PD-1/PD-L1 signaling following IL-15-mediated immune activation (120). These findings provide a strong mechanistic rationale for combining IL-15-based therapies with immune checkpoint blockade, an approach that has already informed the development of several next-generation immunocytokines and combination strategies in cancer immunotherapy.

IL-15-armed adoptive cell therapies represent one of the most promising applications of IL-15 biology. CAR-T, CAR-NK, CAR-NKT, and *γδ* CAR-T cells engineered to express IL-15 or IL-15/IL-15R*α* complexes demonstrate enhanced persistence and superior antitumor activity across hematologic and solid malignancies. Importantly, most studies have not observed autonomous growth or malignant transformation of IL-15-engineered cells, reflecting the localized, low-level production of IL-15 within the tumor microenvironment. Nevertheless, excessive IL-15-driven immune expansion can be hazardous, as illustrated by lethal toxicity in certain AML xenograft models and the transient myelotoxicity observed in CAR-NK clinical trials (74). These data emphasize the need for inducible or spatially restricted IL-15 expression strategies in future cellular therapies.

Another critical consideration is the temporal dynamics of IL-15 exposure. While sustained IL-15 signaling supports CD8^+^ T-cell memory and effector function, chronic IL-15 stimulation of NK cells can induce functional hyporesponsiveness and alter receptor balance (121). Intermittent cytokine delivery (122) or combinatorial priming with IL-12, IL-15, and IL-21 to generate memory-like NK cells (123, 124) may provide strategies to preserve NK-cell potency while avoiding exhaustion. Collectively, future IL-15-based therapies will require precision control over cytokine dose, timing, and localization. Advances in cytokine engineering, immune-cell programming, and biomarker-guided patient selection are expected to enable safer and more effective deployment of IL-15, positioning it as a central component of next-generation cancer immunotherapy.

## 6 Conclusion

In summary, the development of IL-15 as an immunotherapeutic agent has introduced new opportunities beyond traditional cytokine strategies. While further work is needed to refine its safety profile and therapeutic performance in cellular platforms, ongoing innovation is anticipated to fuel meaningful advances in IL-15-based immunotherapies over the coming decades.
